# Influence of prenatal cannabinoid exposure on early development and beyond

**DOI:** 10.3389/adar.2023.10981

**Published:** 2023-02-28

**Authors:** Megan K. Mulligan, Kristin M. Hamre

**Affiliations:** ^1^ Department of Genetics, Genomics and Informatics, University of Tennessee Health Science Center (UTHSC), Memphis, TN, United States; ^2^ Department of Anatomy and Neurobiology, University of Tennessee Health Science Center (UTHSC), Memphis, TN, United States

**Keywords:** development, cannabinoids, prenatal, cannabis, THC

## Abstract

Public perception surrounding whether cannabis use is harmful during pregnancy often diverges greatly from the recommendations of doctors and healthcare providers. In contrast to the medical guidance of abstinence before, during, and after pregnancy, many women of reproductive age believe cannabis use during pregnancy is associated with little potential harm. Legalization and social cues support public perceptions that cannabis use during pregnancy is safe. Moreover, pregnant women may consider cannabis to be a safe alternative for treating pregnancy related ailments, including morning sickness. Compounding the problem is a lack of medical and federal guidance on safe, low, or high-risk levels of cannabis use. These issues mirror the continuing debate surrounding alcohol use and health, in particular, whether there are safe or lower risk levels of alcohol consumption during pregnancy. Clinical studies to date suffer from several limitations. First, most human studies are correlative in nature, meaning that causal associations cannot be made between *in utero* cannabis exposure and health and behavioral outcomes later in life. Due to obvious ethical constraints, it is not possible to randomly assign pregnant mothers to cannabis or other drug exposure conditions—a requirement needed to establish causality. In addition, clinical studies often lack quantitative information on maternal exposure (i.e., dose, frequency, and duration), include a small number of individuals, lack replication of outcome measures across cohorts, rely on self-report to establish maternal drug use, and suffer from unmeasured or residual confounding factors. Causal associations between maternal cannabis exposure and offspring outcomes are possible in preclinical cohorts but there is a large amount of heterogeneity across study designs and developmental differences between rodents and humans may limit translatability. In this review, we summarize research from human and preclinical models to provide insight into potential risks associated with prenatal cannabinoid exposure (PCE). Finally, we highlight gaps in knowledge likely to contribute to the growing divide between medical guidance and public attitudes regarding cannabis use during pregnancy.

## Introduction

Cannabis is the most frequently used illicit drug during pregnancy. Use by pregnant women has been increasing in parallel with legalization of cannabis for medicinal and recreational purposes and public perceptions that use of cannabis products is not harmful. Two large studies of self-reported cannabis use in pregnant women in the US from 2002 to 2017 [467,100 women; National Survey on Drug Use and Health, NSDUH (1)], and in Northern California from 2006 to 2016 [279,457 women; Kaiser Permanente Northern California (2)], reported an increase in cannabis use over time. Past-month use reported by pregnant women in the NSDUH study was 7% in 2016–2017, an increase of 3.6%, while past-month daily/near daily use by pregnant women was 3.4%, an increase of 2.5% (1). Over the course of the Kaiser Permanente study the prevalence of cannabis use by pregnant women increased by 2.9% (3). Notably, cannabis use by pregnant women was highest during the first trimester relative to other trimesters. In the NSDUH study, 11% and 5.3% of pregnant women in 2016–2017 self-reported first trimester past-month use and daily/near daily use, respectively, and lower cannabis use during later trimesters (4).

It is possible that legalization of cannabis for recreational purposes has contributed to increased cannabis use in both pregnant and non-pregnant women. This relationship has not been rigorously evaluated but numerous studies have found an inverse relationship between cannabis use and perceived risk [for review see (5)]. One recent study found evidence in support of an additive interaction between cannabis use and perceptions of cannabis as being low-risk and available (5). Pregnant woman also reported cannabis use to manage or relieve stress, depression, or nausea (6–8). Perceptions of cannabis as safe and having medicinal properties are easy to reinforce through social networks, media, and/or commercial messaging. In one study, researchers contacted dispensaries in the guise of pregnant women suffering from vomiting and nausea, a common first trimester ailment. Out of the 400 Colorado cannabis dispensaries contacted, 69% recommended the use of cannabis products to manage symptoms of morning sickness (9). In another study, over 30% of online media included the use of cannabis to manage nausea and vomiting (10).

In stark contrast to public perception, cannabis use during pregnancy and lactation is strongly discouraged by the American College of Obstetricians and Gynecologists (11), the American Academy of Pediatrics (12), and the Society of Obstetricians and Gynaecologists of Canada’s (SOGC) policy (13). Another growing concern among the medical and research community is that the potency (THC level) of cannabis and derived products has been steadily increasing since the 1990s (14). The impact of maternal consumption of high potency cannabis on fetal development is unknown, but exposure to higher levels of THC may have adverse effects on development. The most common route of cannabis administration reported by pregnant woman is smoking blunts or joints (6). Relative to oral administration, inhalation is associated with faster adsorption and higher THC bioavailability, however, oral administration may result in more prolonged exposure to certain classes of THC metabolites [see (15) for review]. The precise impact of potency and patterns of maternal cannabis use on the developing fetus remains unclear. Regardless of the method of consumption, it is clear from human and preclinical studies that the major psychoactive component of cannabis, THC, crosses the placenta into fetal tissues where it has the potential to interfere with development [for review see (16) and (17–25)].

In this review we provide a brief overview of the endogenous cannabinoid system, its role in development, and possible disruption due to cannabis use. We also summarize research from human and preclinical models to provide insight into potential risks associated with prenatal cannabinoid exposure (PCE). Finally, we highlight limitations of the current research and areas where further research is needed.

## Role of endogenous cannabinoid system in development

Exposure to cannabis during critical periods of development can interfere with homeostatic endocannabinoid system (ECS) function. The ECS plays a critical role in all stages of development, from fertilization, through adolescence, and beyond. Thus, exposure to cannabis has the potential to disrupt ECS function at nearly all stages of life. Below we provide a brief overview of the main components of the ECS and the potential impact of developmental cannabis exposure with a focus on brain development.

The main endogenous ligands of the ECS are the lipids N-arachidonylethanolamide (AEA or anandamide) and 2-arachidonoylglycerol (2-AG). AEA is synthesized from membrane precursors by N-acylphosphatidylethanolamine-specific phospholipase D (NAPE-PLD) and 2-AG is synthesized by 1,2-diacylglycerol (DAG) lipases DAGLα and DAGLβ. AEA is catabolized by fatty acid amide hydrolase (FAAH) whereas 2-AG is catabolized by monoacylglycerol lipase (MGLL). The primary effectors of the ECS are the G-protein coupled receptors (GPCRs) cannabinoid receptor 1 (CB1) and 2 (CB2). AEA has partial agonist activity at the CB1 and less so at the CB2. 2-AG acts as a full agonist at both cannabinoid receptors. AEA and 2-AG can activate other receptors, including GPCRs 55 and 119, and peroxisome proliferator-activated receptor (PPARs). AEA can also act as an agonist at transient receptor potential (TRP) channels (e.g., TRPV2, TRPV3, TRPV4, TRPA1, TRPM8). For a more detailed review see (26).

Direct disruption of the ECS by developmental cannabis exposure is thought to occur primarily through the binding of THC to CB1 and CB2. TRP channels and several orphan G-protein coupled receptors (e.g., GPR55, GPR18) have also been shown to respond to THC and other cannabinoids found in cannabis (e.g., cannabidiol or CBD). However, their role in disruption of the ECS and developmental processes is less well understood. Indirect disruption of the ECS can also occur by altering the levels of endogenous cannabinoids. Very early in development, the endogenous endocannabinoids 2-AG and AEA and their receptors, CB1 and CB2 are alternatively expressed in a delicate spatial and temporal balance in reproductive tissue, uterus, placenta, and in the developing embryo and fetus where they play a collective role in fertilization, implantation, decidualization, and placentation [reviewed in (27)]. Moreover, use of cannabis prior to pregnancy and early in pregnancy (i.e., first trimester) could interfere with ECS homeostasis leading to infertility and adverse outcomes during pregnancy including inhibition of embryonic growth and miscarriage.

The ECS also plays a critical role in fetal brain development later in pregnancy (i.e., second and third trimesters). The binding of THC to CB1 and CB2 is known to disrupt neuronal development and connectivity (28–31). CB1 expression is evident in the developing human brain by 14 weeks and adult brain levels are reached by the end of the second trimester (24 weeks), albeit with regional expression differences apparent between fetal and adult brain (32). It is reasonable to assume that exogenous cannabinoid exposure, especially THC, during this time could interfere with cannabinoid receptor signaling and ECS function. Indeed, modulation of CB1 function during development in preclinical models results in disruptions in axonal pathfinding, progenitor cell expansion and neurogenesis, and specification of neuronal and glial cell lineages (33–35). Moreover, genetic deletion of CB1 in preclinical models is associated with altered morphology or function in numerous brain structures. These include cerebellum (36), cortex (37–39), striatum (40), bed nucleus of the stria terminalis (41), and other mesocorticolimbic areas (42).

Adolescence (12–18 years-of-age in humans and postnatal days 25 through 58 in rodents) is yet another critical period in brain development where the ECS plays a major role in the maturation and plasticity of corticolimbic brain regions [reviewed in (43)]. Adolescence is marked by neuronal circuitry maturation, synaptic remodeling and an overall reduction in synapse numbers, increasing white matter volume, and increasing cognitive capability (44). Prenatal exposure to THC may sensitize or subtly alter neuronal circuits leading to enhanced vulnerability and impairments that appear later in adolescence (45–47). For example, alterations in dopamine D2 (48) and µ opioid (49) receptors have been observed in human fetuses following prenatal cannabis exposure, although the duration of these alterations is unknown. In rodents, long lasting changes in dopamine (48, 50, 51) and opioid brain circuitry (specifically µ opioid receptor levels) have been observed along with increased seeking of heroin in adulthood (52, 53). Changes in adolescent behavior following prenatal THC exposure, including altered activity (54, 55), impaired memory (51, 56, 57), and inhibited social interactions and emotional reactivity (58, 59) have also been reported and will be reviewed in detail in later sections. Taken together, exposure to cannabis during pregnancy has the potential to disrupt the delicate balance of the ECS function and interfere with development. In the following sections we review birth and longer-lasting outcomes associated with PCE based on human and rodent research.

## Clinical birth outcomes associated with PCE

Numerous studies (Table 1) in human cohorts have examined the potential relationship between PCE and birth or perinatal outcomes (i.e., low birthweight, stillbirth, preterm birth, neonatal distress, morphological defects, etc.). As mentioned previously, the ECS is important for implantation, placental development, and maintenance of the pregnancy [reviewed in (60)] and disruption of the ECS through exogenous cannabinoid exposure could exert direct effects on fetal development or indirect effects on intrauterine growth and survival.

**TABLE 1:** Birth outcomes.

Study	Region	Cohort	Exposed	Years	Cannabis use	Maternal use classification	Outcomes
(61)	South Australia	7,301 births	394	1975 to 1981	Self-Report	Nonuser	*Premature birth, fetal growth restriction*
						Up to once per week (356)	
						> once per week (36)	
(62)	Boston, United States	1,690 mother/child pairs	∼230	1977 through 1979	Self-Report	Never	*Low birth weight*
						Once per month (51)	
						Once per month but < once per week (51)	
						1-2 times per week (101)	
						3+ timer per week (34)	
(63)	Connecticut, United States	3,857 mothers	367	1980 through 1982	Self-Report	Nonuser	*Low birth weight, small for gestational age, premature birth (white women only)*
						Occasional user (once per month or less; 158)	
						Regular User (2-3 times per month or more; 209)	
(64)	Ontario, Canada	98,512 women	5,639	2012 to 2017	Self-Report	No	*Preterm birth, low birth weight, placental abruption, transfer to neonatal intensive care, and low Apgar scores*
						Yes	
(65)	8 countries	Meta-analysis of 24 studies	Variable	1982 and 2014	Self-Report	NA	*Low birth weight and transfer to neonatal intensive care*
(66)	Boston, United States	1,226 mothers and children	331	1984 through 1987	Self-Report and Toxicology	Negative	*Low birth weight, decrease in body length*
						Positive	
						(For both self report and urinalysis)	
(67)	7 University prenatal clinics across the United States	7,470 women	822	1984 to 1989	Self-Report and Toxicology	Negative	Cannabis use during pregnancy not related to preterm birth or low birth weight
						Positive	
						(For both self report and serum analysis)	
(68)	Boston, United States	12,424 women	1,246	August 1977 and March 1980	Self-Report	None	Low birth weight, premature birth and major malformations more frequent for cannabis users but these were no longer significant after adjusting for confounding factors
						Occasionally (880)	
						Weekly (229)	
						Daily (137)	
(69)	Avon, United Kingdom	12,129 mothers	585 (use prior to pregnancy); 311 (use in 1st trimester), 250 (use mid pregnancy)	1991 through 1992	Self-Report	Never	After adjusting for confounding factors, no outcomes were significant but there was a trend for negative association between frequency and duration of cannabis use during pregnancy and birth weight
						6 months before pregnancy: Daily (109), 2-4 times per week (149), Once per week (49), <Once per week (278)	
						1st Trimester: Daily (61), 2–4 times per week (84), Once per week (34), <Once per week (132)	
						Mid-pregnancy: Daily (53), 2–4 times per week (59), Once per week (39), <Once per week (99)	
(70)	Norway	9,312 women (10,373 pregnancies)	272	1999 and 2008	Self-Report	None	*Low birth weight*
						Previous use before pregnancy (10,101)	
						Curtailed use during 1 period in pregnancy (209)	
						Prolonged use during at least two periods of pregnancy (63)	
(71)	United States	12,578,557 women and births	66,925	1999 to 2013	Electronic Medical Health Records	No diagnosis	*Intrauterine growth restriction, premature birth, stillbirth*
						Diagnosis of cannabis dependence or abuse	
(72)	New York, United States	139 aborted fetuses	44	2000 to 2002	Self-Report and Toxicology	Non-users	*Reduction in body weight and foot length*
						Before pregnancy: Nonuse (8), Light use (17), Moderate use (4), Heavy use (15)	
						During pregnancy: Nonuse (8^a^), Light use (13), Moderate use (7), Heavy use (10)	
(73)	Rotterdam, Netherlands	7,452 mothers	459	April 2002 to January 2006	Self-Report	Nonuse	*Growth restriction of the fetus in mid- and late-pregnancy and lower birth weight*
						Continued cannabis use (41)	
						Cannabis use in early pregnancy (173)	
						Cannabis use before pregnancy (245)	
(74)	Ohio, United States	325 mothers	111	2010 to 2015	Self-Report and Toxicology	No exposure	*Increased risk of small for gestational age* (*less than 10*th *and 5*th *percentiles*)
						Prenatal cannabis exposure (37)	
						Prenatal	
						cannabis and tobacco exposure (74)	
						Prenatal tobacco exposure (66)	
(75)	Ohio, United States	363 women	119	2010 to 2015	Self-Report and Toxicology	Negative	No association with preterm birth in this at-risk cohort
						Any positive (based on questionnaire, obstetrical record, or urine toxicology)	
(76)	California, United States	3,067,069	29,112	2011 through 2017	Electronic Medical Health Records	No cannabis-related diagnosis	*Premature birth, small for gestational age, admission to neonatal intensive care unit, major structural malformations (brain and gastrointestinal)*
						Any cannabis-related diagnosis	

Italicized text indicates significant associations with PCE. Exposed = Number of individuals within each cohort with cannabis use based on self-report or other measures. Maternal use classification defined in each study with the estimated number of individuals based on available summary tables. Light use = 0 > average joints per day <0.4; moderate use = 0.4 average joints per day <0.89, heavy use = average joints per day ≥0.89.

^a^ self-reported non-use but positive toxicology test.

The most frequently reported adverse birth outcome following PCE in clinical studies (Table 1) is low birth weight (62–66, 70–73, 76). Premature delivery (61, 63, 64, 71, 76) and admission to neonatal intensive care (64, 65, 71, 76) are also frequently associated with PCE. The studies reported in Table 1 often measure multiple birth outcomes, many of which are not significantly associated with PCE. In addition, several studies found no association between PCE and birth outcomes after correcting for potential confounding factors (67–69, 75). Most studies in Table 1 included a modest number (<1,000) of women with exposure to cannabis during pregnancy. A few studies increased this number several fold by leveraging information available through electronic medical health records (71, 76). Digital health information is a useful resource to evaluate the potential impact of PCE in larger populations but generally precludes analysis of cannabis exposure (i.e., frequency and use patterns) during pregnancy and may even select for individuals with heavy cannabis use.

Low birth weight, premature delivery, and admission to neonatal intensive care are the most frequently reported adverse birth outcomes significantly associated with PCE. Stillbirth and gross morphological defects were rarely associated with PCE, which suggests that the range of possible adverse outcomes following cannabis exposure may be relatively narrow. One important consideration affecting most of the studies in Table 1 is the inability to quantify dose, duration, frequency, and amount of exposure. In some studies, maternal exposure is classified by patterns of use (see Table 1), however the numbers in each category are frequently small and classification relies on self-report. Moreover, the composition of cannabis has changed substantially over the duration of these studies. In the US, the amount of THC in cannabis (i.e., potency) has tripled from ∼4% in 1995 to ∼12% in 2014 while the CBD content has decreased from 0.28% in 2001 to less than 0.15% in 2014 (14). In the Netherlands, potency is likely to be even higher with THC levels of 17.7% reported for Dutch cannabis products (77).

Differences in cannabis composition and potency complicate comparisons of outcomes between studies. It is unclear whether the steep increase in cannabis potency is associated with any increases in birth outcome severity (Table 1). This issue has not been extensively explored, partly due to the inability to accurately quantify fetal exposure over the duration of the pregnancy and it remains unclear how potency and level of maternal cannabis exposure contribute to the risk of adverse birth outcomes. It is also important to acknowledge that associations found between cannabis exposure in pregnancy and birth outcomes in Table 1 do not imply causality. Other limitations include a reliance on self-report to assess fetal exposure, different comparison groups and control of possible confounding variables among studies, generally small sample sizes and possible selection bias (i.e., study individuals are not representative of the general population), and potential confounding by unmeasured or residual variables (e.g., socioeconomic status, maternal and fetal genetic factors, maternal behavior and nutrition, polydrug exposure, etc.).

## Long-lasting impact of PCE on behavior and human brain development and function

Given the ubiquitous role of the ECS in development, from fertilization through adolescence, a reasonable hypothesis is that prenatal exposure to cannabis will have a long-lasting impact on child development. Below we summarize the research addressing this assertion. Most of the research into the potential long-term behavioral consequences associated with maternal cannabis use is based on population-based longitudinal cohorts where healthy mothers are recruited from the population and both mothers and offspring followed up at regular intervals.

### Human longitudinal cohorts

The Maternal Health Practices and Child Development Study (MHPCD) and Ottawa Prenatal Prospective Study (OPPS) are the most comprehensive longitudinal studies yet completed. The OPPS (78) cohort primarily consisted of low-risk, middle-class white Canadians and included a total of 49 offspring tracked annually between birth and the age of 6, followed by less frequent follow ups through 22 years-of-age. The high-risk MHPCD (79, 80) cohort consisted of 763 mother-infant pairs at inception and included high-school-educated mothers of lower socioeconomic status and mixed ethnicity (52% African Americans and 48% European Americans) with light-to-moderate use of cannabis, alcohol, and nicotine living in the Pittsburgh, PA area. Cannabis and other drug use was assessed by self-report before pregnancy, at each trimester, and during subsequent follow-up interviews at 8 and 18 months and 3, 6, 10, 14, 16, and 22 years postpartum.

The largest international longitudinal cohort, with ∼100,000 pregnant women recruited between 1998 and 2008, is the Norwegian Mother and Child Cohort Study (MoBa). Biological samples and questionnaire data were collected starting at 17 weeks of gestation. This low-risk and healthy lifestyle cohort has resulted in ∼270 published studies to date. However, as of 2022, only a single study on birth outcomes related to PCE (70) (Table 1) has been published. Additional longitudinal cohorts are in progress.

Other ongoing studies include the Adolescent Brain Cognitive Development (ABCD) study, the Generation R study (GenR), and the Lifestyle and Early Achievement in Families study (LEAF). The largest US cohort is the ABCD study, which consists of ∼12,000 US children enrolled between the ages of 9 and 10 at 21 different sites. The ABCD participants will be tracked for 10 years. The GenR study (81) enrolled 9,778 pregnant women with delivery dates from April 2002 to January 2006. These women were of higher socio-economic status and primarily from Dutch, Surinamese, Turkish and Moroccan ethnic groups in Rotterdam in the Netherlands. Outcomes were measured in early pregnancy (73; Table 1) with some offspring assessed through young adulthood. Behavioral outcomes at birth through age 6 are currently available. The LEAF study population consists of a high-risk cohort of pregnant women (63% African American) enrolled into the Ohio Perinatal Research Repository and includes 362 offspring (116 with prenatal cannabis exposure) eligible for continued follow-up from 3.5 to 7 years beginning in September of 2016 and continuing through August of 2020 (82). Unlike the other cohorts, LEAF study participants provided clinical samples (blood and urine) at enrollment and at each trimester. Thus, cannabis exposure based on THC metabolites can be assessed prospectively for this cohort. Findings from these longitudinal studies are discussed below.

### Cognitive deficits

Cognitive ability and executive function span many dimensions including intelligence, achievement, comprehension, memory, attention, and impulse control. Cognitive outcomes associated with PCE in the various longitudinal cohorts are summarized below and in Table 2.

**TABLE 2:** Alterations in cognitive function related to PCE in human longitudinal cohorts.

Cohort	Infancy (0 to 12 months)	Early childhood (1 to 4 years)	Middle childhood (5 to 8 years)	Preadolescence (9 to 12 years)	Adolescence (13 to 17 years)	Young adult (18 to 22 years)
OPPS	(95, 96) *Poor habituation to visual stimuli, increased tremors and startles (BNAS)* at 4 days old (97). *Increased fine tremors, Moro reflex tremors, startles, heightened motor reflexes (PNE, 9 and 30 days old) and increased hand to mouth behavior* (9 days old)	(98) No adverse effect on mental, motor, visual, or language outcomes (ages 1 and 2, BSID and RDLS) (83). No difference in verbal, quantitative, general cognitive, memory, and motor ability or language comprehension (MSCA/RDLS (age3) (83). *Lower verbal ability and memory subscale associated with heavy prenatal use* (MSCA and PPVT-FL, age 4)	(88) No difference in global intelligence, or cognitive and language ability or memory (aged 5 and 6 years, MSCA and PPVT-FL) (93). No difference on impulse control, deficit in sustained attention (age 6) (93) *Higher parental ratings of impulsivity/hyperactivity* (age 6)	(87) No difference in reading and language measures on comprehensive test battery including WISC-III and WRAT (aged 9–12) (86). No difference in global intelligence or verbal ability but *decreased visual analysis and hypothesis testing and decreased impulse control* (WISC-III, Category Test, GDT age 9–12) (90). No difference in visuoperceptual function (TVPS). *Decreased performance in visual problem solving associated with heavy prenatal use* (WISC-III, age 9–12)	(85) No difference in most tests related to general intelligence, achievement, memory, and executive function. *Deficit in visual memory, analysis, and integration tasks* (age 13 to 16, WRAT, PIAT, MN, AD, SMT, KCT, WCST, Stroop, WISC-III) (89). *Less consistency in reaction times and more omissions indicative of a deficit in attentional stability* (age 13 to 16, CPT, WCST, ST, WISC)	(94) *Higher impulsivity and increased activity in bilateral PFC and right PMC and attenuation of activity in left CB during response inhibition* (age 18 to 22, Go/NoGo, fMRI) (91). No differences in visuospatial working memory (V2B). *More activity in left inferior and middle frontal gyri, left parahippocampal gyrus, left middle occipital gyrus, and left cerebellum. Less activity in the right inferior and middle frontal gyri* (fMRI, age 18–22) (92). No difference in task performance (V2B, Go/NoGo, L2B, CST) *but changes in blood flow during tasks were altered, specifically, increased activity in posterior brain regions* (fMRI, ages 18–22)
MHPCD		(99) *Lower SBIS composite score, lower verbal reasoning and short-term memory deficits* (age 3)	(79) *Lower SBIS composite score, lower quantitative and verbal reasoning and deficits in short-term memory* (age 6) (100). *Higher impulsivity on CPT* (age 6)	(101) *Poor performance WRAT-R reading and spelling scores and PIAT-R reading comprehension score. Higher rate of educational underachievement* (age 10) (102). *SNAP attention deficits, hyperactivity, impulsivity* (age 10) (110). *Deficits in design memory and screen score* (age 10, WRAML)	(103) *Decreased WIAT composite and reading scores* (age 14) (104). *BCT decreased performance on one measure of processing speed and two measures of interhemispheric coordination and better performance on one measure of visual-motor coordination* (age 16)	(105) *Indirect effect on adult memory (WMS-III) mediated through intelligence (age 6), memory (age 10), and early-onset cannabis use (age 22)*
GenR		(106) *Attention problems in females* (CBCL, age 18 months)	(107) *Thicker frontal cortex with no change in gray or white matter volumes* (MRI, ages 6–8)			
LEAF		(108) No difference in executive functioning (age 3.5)				
ABCD				(109) No cognitive deficits or changes in brain activity (fMRI) during tasks measuring response inhibition (SST), reward processing (MID), and working memory (EN-Back). *Higher attention problem score* (CBCL, ages 9–10)		

Italicized text indicates significant associations with PCE. PNE, Prechtl neurological examination; BNAS, Brazelton Neonatal Assessment Scale; BSID, Bayley Scales of Infant Development; RDLS, Reynell Developmental Language Scales; MSCA, McCarthy Scales of Childrens’ Abilities; PPVT-FL, Peabody Picture Vocabulary Test-Form L; SBIS, Stanford-Binet Intelligence Scale; CPT, Continuous Performance Task; MRI, Magnetic Resonance Imaging; WISC-III, Wechsler Intelligence Scale for Children; WRAT, Wide Range Achievement Test-Revised; Category Test, test of abstraction or concept formation ability; GDT, Gordon Diagnostic Delay and Vigilance Task; TVPS, Test of Visual-Perceptual Skills; PIAT-R, Peabody Individual Achievement Test-Revised; SNAP, Swanson, Noland, and Pelham attention subscale; fMRI, functional MRI; SST, Stop Signal task; MID, Monetary Incentive Delay task; EN-Back, EN-Back task of working memory; CBCL, Child Behavioral Checklist; PIAT, Peabody Individual Achievement Test; MN, Missing Numbers test of auditory and visual memory; AD, Abstract Designs test of auditory and visual memory; SMT, Sentence Memory Test of auditory and visual memory; KCT, Knox Cube Test of auditory and visual memory; WCST, Wisconsin Card Sorting Test; Stroop, Stroop Color/Word Interference Test; WIAT, Weschler Individual Achievement Test; BCT, computerized Bimanual Coordination Test; PFC, Prefrontal Cortex; PMC, Premotor Cortex; CB, Cerebellum; V2B, Visuospatial 2-Back task; Go/NoGo, Go/NoGo task; L2B, Letter 2-Back task; WMS-III, Weschler Memory Scale-3rd Edition.

#### OPPS

Major deficits in cognitive function are not consistently detected across developmental stages in the OPPS cohort. A significant association with PCE was not detected until age 4, at which point maternal cannabis use was associated with lower verbal and memory domains assessed as part of the McCarthy Scales of Childrens’ Abilities (MSCA) (83). These associations were no longer significant at later developmental periods following adjustment for potential covariates (84–88). During preadolescence (i.e., ages 9–12), some aspects of visual-motor integration, non-verbal concept formation, and visual problem solving were significantly associated with PCE (86, 89, 90). Some of these PCE associated deficits (e.g., alterations in visual memory, analysis, and integration) persisted through adolescence (85). However, in young adults (i.e., ages 18–22), PCE was no longer found to predict deficits in visuospatial working memory (e.g., Visuospatial 2-Back or V2B) (91). Long lasting alterations in brain function may still be associated with PCE in the absence of profound deficits in cognitive function. Young adults with PCE showed significant and differential activation of brain regions during task performance (e.g., V2B, Go/NoGo, Letter 2-Back or L2B, Counting Stroop Test or CST) as measured by functional magnetic imaging (fMRI) even though task performance was similar between individuals with and without PCE (91, 92).

In contrast, deficits in attention and impulsivity associated with PCE were identified at multiple developmental stages in the OPPS cohort. Sustained attention deficits were significantly associated with PCE at age 6 (93) as were higher parental ratings of impulsivity and hyperactivity (93). During preadolescence, PCE was associated with decreased impulse control (86). During adolescence (i.e., ages 13–17), mild attentional deficits were significantly associated with PCE (89). In young adults, higher levels of impulsivity and alterations in cortical and cerebellar brain activity during response inhibition were significantly associated with PCE (94).

#### MHPCD

Potential cognitive issues related to PCE exposure appeared earlier in development and were more consistently detected for the higher risk MHPCD cohort. At the 3-year follow up during early childhood, maternal cannabis use in the second trimester was significantly and negatively correlated with short term memory subscale scores on the Stanford-Binet Intelligence Scale (SBIS) (99). In African Americans, first trimester use predicted a lower score on the verbal reasoning subscale and second trimester use predicted a lower score on the short-term memory subscale. In European Americans only, preschool attendance counteracted the negative impact of PCE on short-term memory and verbal reasoning. Cognitive deficits persisted at the 6-year follow up during middle childhood and problems of attention and impulsivity emerged. At this age, lower intelligence test composite scores were associated with heavy use (i.e., maternal use of one or more marijuana cigarettes per day) (79). Heavy first trimester cannabis use predicted lower verbal reasoning scores while heavy second trimester use predicted lower composite, short-term memory, and quantitative reasoning scores. Heavy use in the third trimester also predicted lower quantitative reasoning scores. Second trimester cannabis use also predicted higher impulsivity scores (i.e., greater errors of commission on the CPT-3) and, counterintuitively, higher attention scores (i.e., fewer errors of omission on the CPT-3) (100).

However, at the 10-year follow up during preadolescence there appeared to be little impact of PCE on most neurocognitive domains tested [e.g., problem solving and abstract reasoning (computerized Wisconsin Card Sorting test or WCST); learning and memory (WRAML); attention, visuomotor tracking and problem solving (Trail Making, Parts A and B), mental flexibility (Stroop Color/Word Interference Test), psychomotor speed and eye-hand coordination (Grooved Pegboard), attention and mental efficiency (Continuous Performance Test), attention, impulsivity, information processing efficiency and motor control (The Pediatric Assessment of Cognitive Performance Test] (110). Notable exceptions included impulsivity, reading comprehension and educational achievement. Second trimester maternal cannabis use predicted higher impulsivity (albeit near the end of the CPT task in the 3rd trial) (110) and was also a significant predictor of reading comprehension scores, teacher evaluations, and under-achievement (a mismatch between ability and teacher-rated academic achievement) (101).

Access to individual longitudinal data across many behavioral outcomes in the MHPCD enabled advanced statistical approaches to evaluate causal interactions and potential mediation between PCE, study outcomes, and both measured and unmeasured variables. Structural equation modeling (SEM) found that first trimester PCE and school achievement at age 10 were both mediated by child psychological status, which was independent of PCE (101). The indirect impact of PCE on adult memory was also assessed by SEM at the 22-year follow up in young adulthood (105). Although the authors found no evidence of a direct effect of PCE on adult working memory, they reported an indirect effect on adult memory mediated through intelligence (assessed at age 6), adolescent memory (assessed at 10 years), and early-onset (initiation at < 15 years of age) cannabis use.

#### Other cohorts

The GenR, LEAF, and ABCD studies also reported attentional deficits associated with PCE, but few cognitive problems following PCE. In the GenR cohort, PCE was associated with attention problems in 18-month-old females as measured using the Child Behavior Checklist (CBCL) for toddlers (106). The LEAF study was specifically designed to test the influence of PCE on executive function (i.e., inhibitory control, attention, planning ability, cognitive flexibility, episodic memory, processing speed, working memory, visual-spatial ability, and emotional regulation) and aggressive behavior in early to middle childhood (82). However, at age 3.5 there was no difference in executive functioning between children with PCE and unexposed children (108). Likewise, during preadolescence (i.e., ages 9 and 10) in the ABCD cohort, cognitive deficits and changes in brain activity (fMRI) during tasks measuring response inhibition (i.e., the Stop Signal Task or SST), reward processing (i.e., the Monetary Incentive Delay task or MID), and working memory (i.e., the En-Back test) were not associated with PCE (109). However, PCE was significantly associated with higher attention problems (CBCL, ages 9–10) (109).

### Brain morphological changes

Imaging studies in these human longitudinal cohorts are beginning to address whether changes in brain function and morphology during development are associated with PCE. Global brain regional volume was assessed by magnetic resonance imaging scans (MRI) in a subset (i.e., 96 children with prenatal tobacco exposure, 54 children with PCE, and 113 unexposed children) of the GenR cohort during middle childhood (i.e., 6–8 years) (107). In this imaging study, PCE was significantly associated with thicker frontal cortices relative to unexposed controls, while changes in gray or white matter volume were not associated with PCE. These findings suggest that children with PCE may undergo delayed cortical maturation and cortical synaptic pruning, a tantalizing result given that cognitive deficits appeared more prominent at earlier developmental stages in several cohorts (e.g., OPPS, MHPCD). However, the association between PCE and cortical morphology is correlative in nature and needs to be examined more rigorously to establish causality, developmental timing, underlying mechanisms, relationship to behavioral outcomes, and reproducibility.

### Psychopathology and externalizing/internalizing behavioral problems

Aggressive behavior, externalizing behavior, and increased psychopathology have been significantly associated with PCE in several cohorts and are summarized below. However, family environment or genetic factors also appear to contribute to these traits.

#### Externalizing problems

Aggressive behavior (as measured using the CBCL for toddlers) in 18-month-old females was significantly associated with PCE in the GenR cohort (106). Aggressive behavior was also significantly higher in 3.5-year-old children with PCE relative to non-exposed children in the LEAF cohort (108). Numerous behavioral problems (i.e., higher withdrawal symptoms, externalizing behavior problems, and oppositional defiant behaviors) reported by a mother or caregiver were also significantly associated with PCE at age 3.5 in the LEAF cohort (108). Later, in middle childhood (i.e., age 6), PCE was associated with a higher level of hair cortisol concentrations relative to non-exposed children, suggestive of alterations in child HPA-axis function following *in utero* cannabis exposure (111). In the ABCD study, PCE was significantly associated with higher externalizing problems and higher total problem scores (CBCL) (109) during middle childhood (i.e., ages 9 and 10). In the GenR cohort, during both middle childhood and preadolescence (i.e., ages 7 through 10), PCE was associated with offspring externalizing problems (112). However, maternal cannabis use prior to pregnancy and paternal cannabis use were also associated with child externalizing problems suggesting that these associations are not due solely to *in utero* cannabis exposure. Shared familial and/or genetic confounding factors or additional residual or unmeasured confounding factors may have contributed to the observed associations in the GenR cohort (112).

#### Internalizing problems

For the MHPCD cohort, at the 10-year follow up, first and second trimester cannabis exposure was significantly associated with higher levels of depressive symptoms based on child self-report (113). However, a subsequent analysis of combined depression and anxiety symptoms at the 10-year follow up found only a marginal association between PCE and levels of self-reported depression/anxiety symptoms (114).

#### Psychopathology

At the 10-year follow up in the GenR cohort, increased psychotic-like experiences were significantly associated with PCE, maternal cannabis use prior to pregnancy, and paternal cannabis use (115). These associations are highly suggestive of multiple shared etiologies for psychopathology. Psychosis proneness (total score on Prodromol Questionaire-Brief Child Version) was also measured in preadolescents (i.e., age 8.9 through 11) in the ABCD study. Continued use of cannabis after knowledge of pregnancy was significantly associated with increased child psychosis proneness (116) and increased psychotic-like experiences (117) and greater psychopathology (i.e., higher CBCL scores for psychotic-like experiences and internalizing, externalizing, attention, thought, and social problems) (117). In the ABCD cohort, longitudinal analysis of children aged 8.9–13.8 years found that maternal use of cannabis after knowledge of pregnancy was associated with persistent vulnerability to psychopathology during the period of preadolescence (118). PCE was also associated with an increased frequency of psychotic symptoms in young adults from the MHCP cohort (119).

### Sleep alterations

Alterations in sleep following PCE have been reported for different ages in several cohorts. A small sleep pattern sub-study (20 controls and 18 cannabis exposed children) of 3-year olds from the MHPCD cohort found that PCE was associated with frequent nocturnal arousals after sleep onset, more awake time following onset of sleep, and lower sleep efficiency without any change in overall sleep duration or time spent in each sleep stage (120). Analysis of sleep patterns at 3.5 years of age in the LEAF cohort found that PCE was significantly associated with more sleep-related problems based on maternal or caregiver reports (108). A recent analysis of over 11,000 children aged 9–10 years enrolled in the ABCD study (242 with likely continuous cannabis exposure during pregnancy) found a trend between sleep problems (assessed using the Sleep Disturbance Scale for Children) and continued use of cannabis during pregnancy relative to no exposure and cannabis use before knowledge of pregnancy (117). Other studies analyzing ABCD data also found significant associations between PCE and several sleep problem scales (121) and that, relative to unexposed offspring, exposed offspring did not benefit from increased sleep in terms of decreased internalizing (mood) problems (122). It should be noted that mothers with cannabis use before and after knowledge of pregnancy were pooled in the Winiger and Hewitt, and Spechler et al., ABCD studies, and thus the amount, duration, and frequency of cannabis exposure over the duration of the pregnancy was not meaningfully assessed, and there is a strong possibility of unmeasured or residual confounding.

### Substance use

Risk of cannabis use later in life appears to be elevated following PCE. In adolescent and young adults from the OPPS cohort, PCE was associated with both an increased risk of using cannabis and cigarettes, and an increased risk of daily cigarette smoking (123). Moreover, the risk of subsequent cannabis use following PCE was much higher for male offspring relative to females. In the MHPCD cohort, PCE was predictive of early onset cannabis use (EOCU, before the age of 15) and frequency of use at the 14-year follow up (124). Although PCE was not directly associated with cannabis use disorder (CUD) during young adulthood (i.e., 22-years-of-age) in the MHPCD population, path analysis incorporating longitudinal cohort data was used to examine potential pathways between PCE and CUD (125). Two indirect paths were found; one path led from PCE to CUD through EOCU while another led from PCE to CUD through depression symptoms at age 10 and EOCU.

### Summary

Cognitive deficits associated with PCE do not appear to be pervasive across human longitudinal cohorts and seem to appear during specific developmental periods. Relative to other cohorts, the higher risk MHPCD cohort with light to moderate use of cannabis and other substances during pregnancy had more cognitive issues related to PCE exposure. These deficits appeared early in development (prior to age 10) and were more consistently detected during this time. However, it is not possible to assess whether the higher burden of early cognitive deficits associated with PCE in the MHPCD cohort was associated with higher *in utero* cannabinoid exposure or other socioeconomic or environmental factors. Patterns of maternal cannabis use before and during pregnancy were quantified based on self-report in the MHPCD, however associations between use patterns and cognitive outcomes were not always linear. It is not clear whether these results reflect statistical issues or developmentally sensitive periods.

In contrast, attention/impulsivity deficits were more consistently associated with PCE across cohorts and developmental stages. Deficits in attention and impulsivity were often detected during development in the OPPS and MHPCD cohorts and were reported at 18-month-of-age in the GenR study, and during preadolescence (i.e., ages 9–12) in the ABCD cohort. Although associations here are correlative, the combined results across cohorts suggest that PCE may impact brain structures and functions governing attentional processes and impulsivity. Another shared finding across the two most comprehensive longitudinal studies yet completed (e.g., OPPS, MHCDP) was the association between PCE and increased risk of substance use later in life. It will be useful to evaluate offspring substance use patterns in ongoing longitudinal cohorts to see if these associations hold across different study populations, covariates, risk levels, and maternal exposures.

Other findings from human longitudinal cohorts are less clear and point to the need for additional insight and research. For example, it is unclear how PCE influences brain structural changes and sleep during development, largely owing to a lack of data across study populations. Evidence for a role of fetal exposure to cannabis and alterations in aggression, depression, and psychosis appear mixed. Associations between PCE and psychosis and/or externalizing/internalizing traits were detected at different developmental stages across cohorts. However, interpretation of these findings is often complicated because environment, family, and genetic predisposition appear to influence these outcomes as much, or even greater than PCE. Future mechanistic studies in preclinical models and advanced statistical modeling in longitudinal cohorts may be able to dissect complex interactions between PCE, environment, genetics, and their combined influence on behavior.

## Support from preclinical studies

Unlike clinical cohorts, preclinical studies have tight control over cannabinoid composition, dose, duration, and frequency of prenatal exposure and can leverage randomized experimental designs to determine causal associations between exposure and experimental outcomes. As summarized below (Tables 3–5), there is some evidence (based on rodent models) that *in utero* exposure to cannabis or its major psychoactive constituent, THC, is associated with adverse birth outcomes and long lasting developmental and behavioral deficits.

**TABLE 3:** Litter outcomes following cannabis or THC exposure in preclinical models.

Citation	Model	Maternal exposure period	Dose	Vehicle	Controls	Birth outcome
(128)	CR CD1^M^	G6 through G15, daily	THC (5,15, 50, or 150 mg/kg), oral (gavage)	Sesame oil		No effect on maternal weight gain. No effect on prenatal mortality, fetal weight, or gross morphology.
(129)	C3H/HeJ^M^	Exp1: G0 through birth Exp2: PND21 (mother) through birth	CE (40% THC, 45% CBD) at a dose of 20 mg/kg THC, oral	Olive oil		Exp1: Acute CE resulted in sedation and a suppression of sexual activity but mating still occurred. *Mean gestational length increased by 1* *day.* No difference in birth weight. Some loss of pups after birth from unknown causes or cannibalization. Exp2: Decrease in social and sexual behavior but mating still occurred. *Mean gestational length increased by 1* *day.* No difference in birth weight. Some loss of pups after birth from unknown causes or cannibalization.
(126)	Swiss-Webster^M^	G6 through birth, daily	THC (25 or 50 mg/kg), s.c	Sesame oil	Pair-feeding and pair-watering; statistical unit is litter	*Reduced litter size and weight at birth. Dose effect on birth weight.* No group differences in maternal weight gain.
(127)	Balb/C^M^	G5.5 through G17.5, daily	Cannabis cigarette (0.3% THC), inhalation	NA	Urine metabolite analysis	No difference in maternal weight gain. No difference in implantation, litter size, fetal growth, or fetal mortality. *Reduction in fetal weight and higher number of males relative to females associated with cannabis treatment. Decrease in fetal-to-placental weight ratio for males. Reduction in fetal lung, brain, thymus, and liver associated with cannabis treatment*.
(140)	CR Albino^R^	Exp1: E14 through PND21 Exp2: E15 through PND21 Exp3: E6 through E15	Exp1: THC or CME (0.5, 1.5, 5.0 mg/kg), oral Exp2: THC or CME (0.5, 1.5, 5.0 mg/kg), oral Exp3: THC and CE (5, 15, 50 mg/kg), oral	Sesame oil	Cross-fostering (Exp2)	Exp1: Tolerance observed after 3–5 days for all treatment groups along with initial and *transient reduction in maternal weight gain at the 5 mg/kg dose.* No impact on gestational length, fetal mortality, fertility, litter size. No difference in pup body weight or sex ratio at weaning. Exp2: Higher mortality at 5 mg/kg THC. Differences in weight and sex ratios at weaning that were not dose dependent. Exp3: *THC and CME treatment groups showed reduced maternal weight gain*. No fetal abnormalities associated with THC or CME.
(139)	H Wistar^R^	G15 through PND9, daily	THC (2.5–5 mg/kg), oral (buccopharyngeal cannula)	Sesame oil		No change in maternal weight gain. No difference in gestational length, litter size, pup weight gain, or postnatal mortality.
(130)	BSF Long Evans^R^	G3 through birth, daily	CE (10 or 150 mg/kg), oral	Olive oil	Pair feeding	*CE reduced maternal food and water consumption and weight gain*. *Lower birth weight for 150 mg/kg CE dose.* No change in litter size or pup mortality at birth. Postnatal mortality and neonatal weight both increases in the CE group at weaning (21 days) and females had still not caught up with controls by 11 weeks of age.
(131)	HLA Wistar^R^	G2 through G22, daily	THC (15 or 30 mg/kg), oral	Sesame oil	Pair fed (food and water); statistical unit is litter	*Lower initial (i.e., first two doses of 30 mg/kg THC) food and water intake. Less weight gain in THC and pair fed groups relative to ad libitum and naïve controls.* No difference in implantation sites, resorptions, perinatal mortality, litter size, or sex ratio. *Positive linear relationship between higher total mortality (i.e., resorptions + perinatal mortality) and pair fed and THC treatment. Lower male birth weight in pair fed and THC treatment groups relative to controls. Lower female birth weight in THC treatment group relative to controls*.
(141)	H Long Evans^R^	G1 through G22, x2 daily + PND 2 through 10, x2 daily	THC (2 mg/kg), s.c	Ethanol, Tween80, and 0.9% saline (1:1:18)	Food intake recorded	No difference in maternal weight gain. No difference in gestational length. No difference in weight on PND2.
(132)	CR Sprague-Dawley^R^	G6 though G15, daily	THC (25, 50, or 100 mg/kg), s.c	Propylene glycol	NA	Lower maternal weight gain (not dose dependent). *Decrease in E20 fetal weight at the 50 mg/kg dose*.
(135)	CR Wistar^R^	G6.5 through G22, daily	THC (3 mg/kg), i.p	1:18 cremophor:saline	Statistical unit is litter; food intake recorded	No difference in maternal weight gain or food intake. No change in litter outcomes (*i.e*., gestational length, litter size). *Fetal growth restriction: Decreased liver to body weight ratio at birth*.
(133, 134)	CR Wistar^R^	G6 through G22, daily	THC (3 mg/kg), i.p	1:18 cremophor: saline	Statistical unit is litter	No change in maternal food intake or weight gain. No difference in litter size or gestational length. *Fetal growth restriction: Symmetrical fetal growth restriction (i.e., reduction in weight and length); THC exposure associated with decreased birth weight and heart-to-body weight ratio, liver-to-body-weight, and brain-to-body-weight ratio. Altered phenotype in E19.5 placenta* (*i.e.*, reduced fetal to placental weight ratio, increased labyrinth layer area and reduced expression of labyrinth progenitors, vascular defects).
(136, 137)	Wistar^R^	G1 through G19	CE (THC ∼3.3 mg), smoke inhalation (filter-tipped cigarette)	NA	Cross-fostering; statistical unit is litter	*Lower birth weight.* No difference in litter size, gestational length, sex ratio, live births, and morphology at birth
(142, 143)	CR Sprague-Dawley^R^	G5 through G20, daily	THC (100 mg/mL at 2 L/min airflow) inhalation (e-cigarette)	Propylene glycol	THC metabolites measured during pregnancy; food and water intake recorded	No difference in maternal weight gain or food and water intake. No change in litter outcomes (*i.e*., gestational length, litter size, sex ratio, or birth weight).
(138)	S Long Evans^R^	G1 through PND2, x2 daily	CE (400 mg/mL) inhalation (e-cigarette)	80% propylene glycol/20% vegetable glycerol	Cross-fostering (most litters contained pups from all conditions)	No difference in litter size. *On PND 6, 10 and 13 air exposed offspring weighed more than CE and vehicle exposed offspring and CE exposed pups weighed more than vehicle exposed pups*.

M, mice; R, rats; BLO, Biobreeding Laboratories Ottawa; BSF, Blue Spruce Farms; CR, Charles River; H, Harlan; HLA, Hilltop Lab Animals; J, The Jackson Laboratory; S, Simonsen Laboratories; s.c., subcutaneous; i.p., intraperitoneal; Exp, experiment; italicized text indicates significant maternal effects; italicized text indicates significant litter outcomes.

**TABLE 4:** Behavioral changes following developmental THC exposure measured in rodents.

Citation	Species/Strain	THC dose & route	Time of admin	Behavior paradigm	Age examined	Outcomes	Sex effects
(164)	Mice: CB1 Conditional KO	3 mg/kg IP inj.	E10–E17	Spatial Memory	P60	Males Impaired Memory	Males
				Object Recognition	P60	No Diff.	No Diff.
(152)	Rat: Wistar	5 mg/kg SC inj.	E5–E20	Social interaction	Adult	Males Decreased Interaction	Males
				Anxiety-like Behavior	Adult	No Diff.	Not Tested
				Cognition	Adult	No Diff.	Not Tested
(33)	Mice: Serotonin Reporter	5 mg/kg IP inj.	E10–E18	Social Interaction	Adult	Decreased Interaction	Not Tested
(172)	Mice: CB1R KO	3 mg/kg IP inj.	E12–E16	Fine Motor Tests	10 weeks	Decreased Fine Motor	Not Tested
(173)	Rats: Sprague Dawley	20 ng/ml Vape	E5–E20	Motor Development	P12–P20	Delayed Capability	No Diff.
				Motor Coordination	P30–32	Poorer	No Diff.
				Activity Level	P31–34	More Activity	Males
				Anxiety-Like Behavior (Center vs. Edge)	P31–34	No Diff.	Males
(174)	Rats: Sprague Dawley	100 mg/ml Vape	E5–E20	Sensori-Motor Development	P12–P20	No Diff.	No Diff.
				Motor Coordination	P30–32	No Diff.	No Diff.
(154)	Rats: Long Evans	0.15 mg/kg IV inj.	E5–P2	Rewarding Behavior	P62	Increased Motivation	Study Includes Males Only
				Depression-like Behavior	P62	Enhanced Depression-like Phenotypes	Study Includes Males Only
(153)	Rats: Sprague Dawley	2 mg/kg SC inj.	E5–20	Locomotor Activity	P28–40	No Diff.	Study Includes Females Only
				Risk Taking	P28–40	No Diff.	Study Includes Females Only
				Anxiety-like Behavior	P28–40	No Diff.	Study Includes Females Only
				Social Behavior	P28–40	No Diff.	Study Includes Females Only
				Anhedonia	P28–40	No Diff.	Study Includes Females Only
				Emotional Memory	P28–40	No Diff.	Study Includes Females Only
(160)	Rats: Sprague Dawley	5 mg/kg Oral	E15–P9	Neonatal Reflexes	P1–P11	Delay in Reflex Development	Not Tested
				Locomotor Activity	P100	No Diff.	Not Tested
				Social Behavior	P100	Decreased Interactions	Not Tested
				Cognitive Behavior	P100	Altered	Not Tested
(156)	Rats: Sprague Dawley	2 mg/kg SC inj.	E5-E20	Depression-like Behavior	Prepuberty	Altered	Study Includes Males Only
				Sensorimotor Gating	Prepuberty	Altered	Study Includes Males Only
(138)	Rats: Long-Evans	2 doses: 400 mg/ml 50 mg/ml Vape	Prior to mating up to P10	Ultrasonic Vocalization	P6, P10, P13	Increased at P6	No Diff.
				Social Play Behavior	P26	Decreased	Males (Some Measures)
				Anxiety-like Behavior	P27	No Diff.	No Diff.
				Adult Anxiety-like Behavior	P73	Increased Anxiety-like Behavior	No Diff.
				Behavioral Flexibility	Adult	Increased Errors (High Dose Only)	No Diff.
(145)	Rats: Wistar	2 mg/kg SC inj.	E5–E20	Locomotor Activity	P25 and beyond	Increased	Not Tested
				Learning & Memory	P25 & Beyond	No Effect	Not Tested
				Aversive Limbic Memory	P25 & Beyond	Impaired Performance	Not Tested
				Motivation For Alcohol (Operant)	P25 & Beyond	Increased Motivation	Not Tested
				Nociception	P25 & Beyond	No Diff.	Not Tested
(151)	Rats: RjHan: Wistar	2 mg/kg SC inj.	P1–P10	Ultrasonic Vocalization	P9, P15	Altered (Both Ages)	Not Tested
				Homing Behavior	P10, P13	No Diff.	Not Tested
(162)	Rats: RjHan: Wistar	2 mg/kg SC inj.	P1–P10	Locomotor Activity	Adult	No Diff.	No diff.
				Social Interaction	Adult	Enhanced Interaction, Diminished Social Memory	No Diff.
				Memory	Adult	No Diff.	No Diff.
(161)	Rats: Sprague Dawley	5 mg/kg Oral	E15–P9	Social Interaction	P180	Decreased interaction	Study Includes Males Only
				Memory	P180	Impaired (Decreased Retention)	Study Includes Males Only
(56)	Rats: Sprague Dawley	0.15 mg/kg IV inj.	E1–E21	Passive Avoidance	P22	Impaired retention	No Diff.
				Active Avoidance	P45	Impaired Reversal	Males
				Attention	P60	Impaired	No Diff.
				Amphetamine Challenge	P60	Dampened Response	No Diff.
(139)	Rats: Wistar	2.5 or 5 mg/kg Orally through Cannula	E15–P9	Ultrasonic Vocalization	P12	Increased (High Dose)	Not tested
				Social Interaction	P35	Decreased Interactions	Not Tested
				Anxiety-like Behavior	P80	Increased Anxiety-like Behavior	Not Tested
(141)	Rats: Long Evans	2 mg/kg SC inj.	E1–E22, P2-10	Activity level	P90	No Diff.	Study Includes Males Only
				Anxiety-like Behavior	P90	Increased Anxiety-like Behavior	Study Includes Males Only
				Social Interaction	P90	Increased Interaction	Study Includes Males Only
				Depression-like Behavrior	P90	No Diff.	Study Includes Males Only
(169)	Rats: Wistar	5 mg/kg Orally through cannula	E15–P9	Inhibitory avoidance	P80	Increased Avoidance	Study Includes Males Only
				Social Discrimination	P80	Impaired Discrimination	Study Includes Males Only
(53)	Rats: Long Evans	0.15 mg/kg IV inj.	E5–P2	Heroin Self-administration	P62	Increased Self-Administration	Not Tested
				Stress-Induced Heroin Administration	P62	Enhanced Administration	Not Tested
				Heroin-Induced Locomotor Activation	P62	Decreased Activity	Not Tested
(148)	Rats: Wistar	5 mg/kg IP inj.	P4–P14	Heroin-induced place conditioning	P56	Increased Preference	Not tested
				Heroin-Induced Locomotor Activation	P56	Effects Observed at Low Dose of Heroin	Not Tested
(175)	Rats: Wistar	5 mg/kg SC inj.	P4–P14	Spatial Discrimination	P56	No Diff.	Study Includes Males Only
				Delayed Alternation	P56	Delayed Acquisition	Study Includes Males Only
(150)	Rats: Wistar	5 mg/kg Oral	E5–P24	Morphine self-administration	P70	No Diff.	No THC -induced diff.
(146)	Rats: Wistar	1 or 5 mg/kg	E5–P24	Morphine conditioned place preference	P70	Enhanced Preference	Males (Both Doses) Females (Low Dose)
				Anxiety-like Behaviors	P70	Less Anxiety	Males
(52)	Rats: Wistar	5 mg/kg Oral	E5–P24	Morphine self-administration	P70	Enhanced Administration	Females
(147)	Rats: Wistar	5 mg/kg Oral	E5–P24	Open Field Behavior	P70	Increase in Behaviors	No Diff.
				Locomotor Activity	P70	Increased Activity	Females
				Morphine Place Preference	P70	Enhanced	No Diff.
(149)	Rats: Wistar	5 mg/kg Oral	E5–P24	Opioid Withdrawal After Naloxone Challenge	P24	Increased Symptoms	Males
				Morphine-Induced Analgesia	P70	Increased Analgesia	Males
(155)	Rats: Wistar	5 mg/kg Oral	E5–P24	Development of motor behaviors over time	P15, P20, P30, P40, P70	Altered on Specific Days	Sexually Dimorphic Based on Day
				Stress-Induced Motor Behaviors	P70	Altered	Females
(144)	Rats: Fischer-344	10 mg/kg Inj.	P4, P6, P8	Stress-induced tail withdrawal	P130	Increased Withdrawal Latency	Not tested

**TABLE 5:** Behavioral outcomes following prenatal polysubstance exposure.

Author	Co-exposure	Exposure parameters	Behavior tested	Thc only effect	Interaction	Sex effect
(173)	THC + Ethanol	GD 5–20 (rats) THC (100 mg/ml; 30 min vapor Inhalation at airflow rate of 2L/min in e-cigarette tank); Ethanol (BAC = 150 mg/dl; vapor inhalation)	Motor Development	Delayed Capability	Enhanced	No
			Motor Coordination	Poorer	No	No
			Activity Level	More Activity	Increased	Males
			Anxiety-like Behavior (Center)	No	Increased Time in Center	Males
(174)	THC + Nicotine	GD 5–20 (rats); THC: 100 mg/ml; Nicotine: 36 mg/ml; 40 min vapor inhalation at airflow rate of 2L/min in e-cigarette tank	Sensori-Motor Development	No	Delayed Success	No
			Motor Coordination	No	Lower Success Rate	Females
(176)	THC + Nicotine	Through Gestation (rats); THC (Oral, Edible); Nicotine (Vapor Inhalation)	Sensori-Motor Gating	No	Deficits	Males
			Memory	Yes	Males (short-term memory deficit with THC + Nicotine)	Males (short-term memory deficit with THC or THC + Nicotine) Females (short-term memory deficit with THC alone)
			Anxiety-Like Behaviors	Increased Anxiety-Like Behaviors	No	Males

Animal models complement clinical studies and provide insight into the range of aberrant offspring outcomes and potential underlying molecular mechanisms following different levels of PCE. For animal models to provide valuable information, one of the important considerations is the comparison between the timing of exposure in the model organism and determining the comparable time in human brain development. Many studies have undertaken these comparisons and identified when specific developmental events, such as the beginning of cortical neogenesis, happen across species. It is now well-established that the first trimester, in terms of brain development, extends until embryonic day (E) 11–13 in mice and E12-15 in rats while the third trimester equivalent happens entirely postnatally in both species. Thus, a host of exposure paradigms have been used that encompass varying epochs in brain development.

To maximize translational relevance in the following review of preclinical studies, this review only reports findings where cannabis/cannabinoid exposure approximates the level of exposure that could reasonably be expected in pregnant women (e.g., smoking cannabis three times per day or less). In addition, only studies that used THC or cannabis extracts (CEs) were included. Similar to the situation in humans, there are several important considerations when interpreting the preclinical findings reported below. First, maternal cannabis exposure has the potential to influence both maternal nutrition (i.e., involuntary exposure may cause decreased food and water intake) and maternal care. Second, the route of administration used to deliver cannabinoids may influence the rate of THC absorption and fetal exposure. Third, the vehicle used to deliver THC, CEs, or cannabis may contain components that produce maternal or offspring developmental effects. Finally, as mentioned above, the duration of exposure and developmental stage is not uniform across preclinical studies. To address some of these caveats we have included information in the tables regarding inclusion of any experimental controls to address maternal cannabis exposure, vehicle composition, duration of exposure, dose, and route of administration. Note that some controls, such as cross fostering, may have complicated consequences on maternal care and pup development.

### Birth outcomes associated with PCE in rodents

There is abundant heterogeneity in the experimental design of preclinical PCE studies (Table 3). Despite this variability, a reduction in birth weight associated with PCE is consistently reported across both mouse and rat studies and this result is also commonly replicated in human studies (Table 1).

In mice there was a marginal relationship between THC dose and litter outcomes. In Swiss Webster and Balb/C mice, daily administration of 25–50 mg/kg THC (*s.c.*) or daily exposure to cannabis cigarettes (5 min exposure to 200 mg cannabis cigarette with 0.3% THC content, inhalation) resulted in a reduction of fetal/birth weight (126, 127). However, daily oral administration of THC or cannabis extracts from 5 to 150 mg/kg had no effect on birth weight in CD1 and C3H/HeJ mice (128, 129). Moreover, litter size, sex ratio, fetal mortality, gestational length, and gross morphology were generally unaffected by PCE at the range of doses reported (Table 3). The exceptions being that a daily oral dose of 20 mg/kg THC slightly increased gestational length in C3H/HeJ mice (129) and exposure to cannabis cigarette smoke altered litter sex ratios in favor of males and contributed to fetal growth restrictions in male treated Balb/C mice (127).

In rats, perhaps owing to a greater number of studies, there was an apparent linear relationship between THC dose and litter outcomes. In Long Evans, Wistar, and Sprague Dawley rats, lower birth weight was associated with daily *in utero* exposure to 15, 30, or 150 mg/kg (oral) cannabis extracts (130, 131), 50 mg/kg (s.c.) THC (132), 3 mg/kg (i.p.) THC (133–135), or smoke from cannabis extracts (400 mg/mL) (130, 136–140). In contrast, no effect of PCE on birth weight was observed following daily *in utero* exposure to 0.5–10 mg/kg (oral) THC or CE (130, 139, 140), 2 mg/kg (s.c.) THC (141), or smoke from CEs (100 mg/mL) (142, 143). In addition, placental alterations were associated with daily *i.p.* injections of THC (3 mg/kg) (134). Litter outcomes did not appear to be strongly influenced by differences in maternal food and water intake or weight gain following cannabinoid exposure (Table 3).

Taken together, preclinical studies in both mice and rats support the major clinical finding of reduced birth weight and fetal growth restriction associated with PCE (Table 1). Because cannabinoid composition and dose can be well controlled in preclinical studies, an additional finding with relevance to human studies is that there is a strong negative effect of THC dose on adverse outcomes. In preclinical rat studies this holds true regardless of the route of administration. For example, exposure to THC above 10 mg/kg (oral), 2 mg/kg (s.c.), or 100 mg/mL (inhalation) in rats resulted in adverse birth outcomes. There are a smaller number of studies in mice and the impact of route of administration and THC dose is less clear, perhaps due to genetic differences among strains or experimental paradigms. More work is needed in mice to evaluate the impact of THC dose and route of administration on litter outcomes. Taken together, preclinical studies in rodents suggest that exposure to higher levels of THC increases the risk of adverse birth outcomes.

Exogenous cannabinoid exposure has the potential to disrupt reproductive processes and alter mating behavior. For this reason, many preclinical studies delay cannabinoid treatment until G5 or G6 in the hopes of increasing the odds of pregnancy. Several studies varied maternal cannabinoid exposure to quantify changes in fertility, productive mating, and implantation (129, 131). However, there appeared to be little effect of low to moderate cannabinoid exposure (15–30 mg/kg, oral) on these outcomes. This is an important consideration as most human mothers consume cannabis prior to pregnancy and during the first trimester with use tapering off in later trimesters (4). Use throughout pregnancy is less common and may be associated with higher risk pregnancies and other maternal characteristics that could influence birth outcomes and later child development. In rodents, the third trimester equivalent occurs outside of the mother’s body. Many of the studies in Table 3 included daily exposure to cannabinoids from G6 through G22, which corresponds to the first and second trimesters in humans. In the rodent studies, daily first and second trimester PCE was often sufficient to cause adverse litter outcomes (i.e., low birth weight).

### Long-lasting impact of PCE on behavioral and molecular outcomes in rodents

Preclinical studies in rodents have provided additional insight into the effects of PCE on a wide range of traits. Examination of the effects of cannabis on brain development began in the 1970s and 1980s [e.g., (144)]. These studies gave the first clues on the types of effects that cannabis had on the developing brain and some studies showed that similar types of behavioral effects were observed in animal models and human populations. With the legalization of cannabis for recreational and/or medical use in many parts of the US, there has been a resurgence in research on its effects on brain development. Similar to what is observed in human populations, the results are not always consistent across preclinical studies. This is likely due to variations in a range of experimental parameters including differences in doses, time of exposure, time of evaluation of the behavioral phenotype, and testing parameters. However, even with these differences, it is clear that a number of different effects have been observed in animals following PCE and the findings are summarized below.

#### Substance use

One of the most consistent findings is that prenatal exposure to THC alters the behavioral response to other drugs of abuse, notably opioids, taken later in life. In these experiments, animals are exposed to THC during prenatal or early postnatal periods followed by exposure to a different drug of abuse during adolescence or adulthood [e.g., (52, 53, 56, 145–149)]. These studies have demonstrated that PCE can influence the rewarding/reinforcing properties and behavioral or physiological responses to other drugs later in life (Table 4). Only a single study failed to show differences between animals with PCE and unexposed controls (150). These results suggest long lasting effects of PCE that may enhance the propensity for substance use disorders later in life.

#### Externalizing/internalizing behavioral problems

Emotional reactivity has been examined in a host of behavioral paradigms and over varying ages. Several studies have examined ultrasonic vocalizations in pre-weanling pups, which are defined as cries from the pup to the dam and interpreted as a sign of distress. The results have shown that THC-exposed pups show altered vocalizations, at least at certain ages, supporting the hypothesis that developmental THC exposure can alter early emotional responding (138, 139, 151). Numerous studies have examined other responses later in life with mixed results. For example, anxiety-like phenotypes have been examined and results have shown increased anxiety-like behavior (138, 139, 141), decreased anxiety-like behavior (146), and no difference from controls (152, 153). Stress reactivity and depression-like phenotypes are not as well-studied. Prenatal THC exposure has been shown to alter both phenotypes in some studies (144, 154–156), while others have shown no significant effects (141, 153).

#### Psychosis

In recent years, it has been proposed that PCE may be a risk factor for psychosis later in life as part of a “two-hit” model [see (157) for review]. The proposed mechanisms behind this model state that prenatal exposure to THC alters the cannabinoid system during development. The second hit happens when an additional environmental exposure occurs, such as stress or THC exposure later in life, that further disrupts the endocannabinoid system ultimately leading to psychosis. Psychosis and Schizophrenia are uniquely human disorders but some aspects of the disease (i.e., endophenotypes) can be studied in rodents. One such endophenotype is altered sensory gating evaluated using the pre-pulse inhibition paradigm or PPI [see (158) for review]. In one of the first studies to evaluate this (159), rats were exposed to THC *in utero* and later during adolescence. The results showed altered sensorimotor gating effects in animals exposed to THC both *in utero* and during adolescence compared to those exposed only *in utero,* only at adolescence, or unexposed controls. Interestingly, the results were observed in males but not in females suggesting some sex-specificity of the effects. These types of sensorimotor gating effects have been replicated in further experiments [e.g., (156)] suggesting the importance of further evaluating this relationship.

#### Social interactions

Mice and rats are highly social animals and have often been evaluated for the frequency and type of social interactions. Social behavior has been evaluated across multiple paradigms. For many of these studies, prenatal THC exposure has been shown to alter social interactions (33, 138,141,152,160,161,162). In contrast, a study by Traccis and colleagues (153) failed to show an effect of prenatal THC on social behaviors.

#### Brain regions and signaling pathways

One advantage of preclinical studies is the ability to dissect the underlying mechanisms associated with changes in behavior following PCE. Although a detailed analysis of current studies is beyond the scope of this review, we touch on a few findings that highlight the utility of rodent models in dissecting the functional implications of changes in brain signaling systems following PCE. Long-lasting alterations in brain reward regions (i.e., the mesolimbic dopamine system including the ventral tegmental area and nucleus accumbens) have been reported following PCE. Changes to this system may mediate enhanced propensity for drug seeking/reinforcing behavior in rodents and risk of substance use and abuse in humans. In rats, PCE is associated with altered dopaminergic function in the ventral tegmental area (159, 163) and decreased expression of dopamine receptor genes (i.e., dopamine receptor D2) in the nucleus accumbens (48). Decreased levels of dopamine receptor D2 mRNA in nucleus accumbens following PCE have also been observed in human fetal tissue (48).

Alterations in cortical and hippocampal regions following PCE could mediate changes in cognition, memory, attention, and impulsivity. Changes in the number or function of hippocampal inhibitory neurons (33, 164, 165) and alterations in both inhibitory hippocampal GABAergic transmission (166) and excitatory hippocampal glutamatergic neurotransmission (167) have been reported following PCE in rats. Prenatal or perinatal exposure to THC or cannabinoid agonists have also been associated with changes in cortical synaptic plasticity and excitatory glutamatergic signaling (51, 168, 169). These are just a few of the many rodent studies that are beginning to quantifying precise molecular and functional perturbations to brain signaling pathways following PCE [for review see (170, 171)].

### Sex, genetic background, and other variables

In addition to confirming results seen in human populations, animal models have also extended these findings particularly by evaluating other variables. One of the most notable examples is the effects of sex. As seen in Table 4, several studies have shown sex-specific effects, although there are a number of studies in which only one sex was evaluated leaving open the question of sex-specific effects. This is clearly an issue that warrants further study in human populations which will likely be facilitated by characterization of additional populations. Surprisingly, the role of genetics in modulating the potential teratogenic effects of THC have been minimally explored, at least as it relates to behavioral effects. Most of the experiments have focused on examination of specific components of the THC pathway, such as the CB1 receptor, using knock out mice [e.g., (164, 172)]. This is also an issue that warrants additional study. Moreover, the role of other variables, such as nutrition or exposure to stress, need additional investigation.

### Prenatal polysubstance exposure

Preclinical models are also invaluable for separating the effects of THC from that of other drugs. The rate of maternal polydrug use during pregnancy is high making it difficult to assess whether effects are due to THC exposure or to exposure to other substances of abuse. As discussed previously, data from preclinical studies demonstrates the potential teratogenic effects of THC on a range of outcomes. However, there can also be interactions among drugs, either in positive or negative directions, and the nature of these interactions can be evaluated in preclinical models.

One of the most commonly co-abused substances is ethanol. Ethanol has been shown to be highly teratogenic with wide-spread effects in many CNS regions and on many developmental processes. Behaviorally, the effects of ethanol exposure have been shown to have some similarities with those of THC including effects on anxiety- and depression-like phenotypes. As shown in Table 5, studies are beginning to evaluate co-exposure to both ethanol and THC (173). While the results demonstrate that each substance alone can cause behavioral alterations, there is also evidence for an interaction of the two drugs suggesting that the co-exposure can worsen the teratogenic effects of each alone.

A second drug with high rates of co-exposure is nicotine either through traditional cigarettes or through e-cigarettes. While limited in nature, studies are beginning to evaluate this co-exposure as well (Table 5). Similar to what was observed for ethanol, different effects of either substance alone or after combined exposure have been found (174, 176). Pronounced sex effects were also observed, suggesting that sex may be a variable in the response to the co-exposure of these two drugs of abuse. Additional work is needed to further expand this interaction.

### Summary

Preclinical rodent models are beginning to quantify the teratogenic profile of cannabis and THC on offspring outcomes following PCE. In these studies, outcomes are specifically associated with *in utero* cannabinoid exposure rather than exposure to other substances of abuse or uncontrolled environmental factors. However, there are limitations to these studies. These include heterogeneity in experimental design (e.g., variation in methods of exposure, dose, cannabinoid, timing of exposure) and phenotypic outcomes measured. Further, because the level of brain development in a newborn rodent differs from that in a newborn human, translating across species can be difficult. Even with these limitations, there are consistent findings across studies on a range of phenotypes including lower weight at birth, altered emotionality, and altered responses to and enhanced propensity to consume other drugs of abuse later in life. Significant associations between PCE and other behavioral deficits have been reported, although the results are not always consistent across studies. Further work is needed to resolve these differences. Moreover, there are numerous issues that are difficult to address in clinical studies that still can be explored further using rodent PCE models. First, it is still unclear whether there are levels of maternal cannabis use that might be associated with less risk to child development. It is also unclear how cannabis composition, especially potency, might influence the risk of adverse birth outcomes or child developmental problems later in life. Second, the contribution of genetic background and/or environmental variables to the susceptibility of teratogenic effects following PCE remain understudied and unclear. Finally, we still know relatively little about the underlying molecular mechanisms and changes to brain structure and function impacted by PCE. Preclinical models are beginning to address the underlying molecular changes associated with PCE and have implicated several neurotransmitter systems, brain regions, and cell types of interest but more work is needed to evaluate structural and functional changes following PCE. This knowledge will be needed to design and evaluate interventions that ameliorate the teratogenic effects of cannabis, particularly as they relate to psychosis risk.

## Discussion

Maternal cannabis use and cannabis potency are both increasing despite the possible adverse and long-term consequences of PCE on child development. However, research to date has yet to establish clear links between adverse offspring outcomes and the frequency, duration, and types of maternal cannabis exposure. Longitudinal studies in humans are important for tracking potential long-lasting outcomes associated with prenatal cannabis exposure. However, all studies to date suffer from small sample sizes, potential confounding factors, outcome measures that differ between studies, and the inability to quantify cannabinoid exposure during pregnancy. Moreover, the prospective design and a lack of randomized conditions in human study cohorts precludes causal inference between *in utero* cannabis exposure and later health and behavioral outcome measures.

Despite these limitations, clinical cohorts and longitudinal studies are beginning to illuminate some of the potential consequences of PCE on offspring outcomes. These include adverse birth outcomes (e.g., low birth weight, premature delivery, admission to neonatal intensive care) and longer lasting behavioral alterations in offspring with PCE (e.g., attention and impulse control deficits, elevated risk of substance use disorders, and a possible increase in psychopathology, aggression, anxiety, and depression). In preclinical models, cannabinoid exposure and environmental conditions can be tightly controlled, and randomized study designs can be applied to infer causality. Moreover, intervening molecular pathways from PCE to phenotypic outcome can be identified as can variables (e.g., exposure level, sex, genotype, environment) that influence outcome severity. Studies leveraging rodents lend support to the hypothesis that human PCE causes a reduction in birth weight and fetal growth restriction, with a longer-term impact on offspring behavior across multiple domains related to substance use, emotional reactivity, and psychopathology.

Much work is still needed to characterize the full spectrum of teratogenic effects associated with different levels of *in utero* exposure to cannabis and cannabinoids. Moving forward, study designs in rodents will need to better model human patterns of cannabinoid exposures, especially variation in cannabis potency and composition, routes of administration, polysubstance interactions, and maternal pre- and post-conception exposures. Clarification of the range and types of offspring phenotypes impacted by PCE, enhanced translational relevance of phenotypes, and better replication of comparable outcomes across studies will be required for clinical and preclinical studies. Rodent studies may be especially useful to disentangle the role of confounding factors (e.g., sex, genetics, multi-drug interactions, and other environmental variables) that have been difficult to model in human studies where sample sizes can be small relative to the number of potential covariates. Finally, the addition of developmental brain molecular, functional, and structural data has the potential to bridge the gap between PCE and behavioral outcomes across species. Rodent studies are beginning to resolve the role of discrete signaling pathways and brain regions in modulating behavioral outcomes following PCE. However, functional and structural imaging studies of brain development following PCE are lacking in humans and rodents. Ultimately, an important goal of future research is to clearly define developmental processes vulnerable to PCE and provide clinicians and patients with more detailed information about specific risks to the child posed by different levels of maternal cannabis exposure.
